# The Rhythms of the Moon: Can the Moon Affect Cardiac Arrhythmias?

**DOI:** 10.19102/icrm.2025.16034

**Published:** 2025-03-15

**Authors:** Athanasios Ziakos, Armin Sause, Melchior Seyfarth

**Affiliations:** 1 Helios Heart Center Wuppertal, Department of Cardiology, University of Witten/Herdecke, Wuppertal, Germany

**Keywords:** Atrial fibrillation, cardiac arrhythmias, lunar phases, moon phases, moon–Earth distance

## Abstract

Diagnosing paroxysmal cardiac arrhythmias early poses a challenge, yet it holds paramount significance. Certain patients hold strong beliefs regarding the moon’s impact on cardiac arrhythmias. This study aims to examine the potential correlation. In our emergency room, each patient presentation is assigned an “admission diagnosis.” An analysis was conducted on admission diagnoses from 2012–2020 (before the coronavirus disease 2019 pandemic). The frequency of rhythmological diagnoses was investigated, both collectively and separately, as well as categorized by the underlying pathomechanism, in relation to the lunar phase and the moon’s proximity to the Earth at the time of admission. Moreover, the impacts of sex, age, and weight were evaluated. A total of 58,230 patient presentations were recorded, with 16.9% coded with rhythmological diagnoses. No significant differences were found in the distribution of cardiac arrhythmias concerning lunar phases or the moon–Earth distance. Sex, age, and weight did not influence this distribution, except in a small group of underweight patients (<55 kg), where a statistically significant difference was observed with greater moon distance. To verify this result, we investigated all existing Holter records of underweight patients presenting to the emergency room between 2017 and 2020. In 195 24-h Holter recordings, a uniform burden of supraventricular extrasystoles and atrial fibrillation/flutter irrespective of the moon’s distance from the Earth was observed. Contrary to patients’ beliefs, the moon does not seem to affect the presentations with rhythmological complaints and diagnoses in our single-center analysis, irrespective of age, sex, or the arrhythmia type. The moon cannot aid in diagnosing paroxysmal arrhythmias.

## Introduction

Most cardiac arrhythmias are paroxysmal, making their diagnosis challenging over extended periods and potentially leading to complications, such as ischemic stroke in atrial fibrillation. Improved timing of diagnostic tools, such as electrocardiography, Holter monitoring, or electrophysiological studies, could facilitate an early diagnosis. Exploring a potential correlation between the incidence of cardiac arrhythmias and lunar phases or the moon’s distance from the Earth could aid in optimizing the arrangement of diagnostic tools. Some patients strongly believe in the moon’s influence on cardiac arrhythmias. This study aims to investigate whether such a correlation exists.

## Methods

At our heart center emergency room (ER), every patient presentation receives an “admission diagnosis” assigned by a doctor. All admission diagnoses from 2012–2020 (before the coronavirus disease 2019 pandemic) were analyzed. The incidence of rhythmological diagnoses (collectively, separately, and in groups based on the underlying pathomechanism) was examined concerning the lunar phase and the moon’s distance from the Earth on the day of admission. Additionally, the influence of sex, age, and weight was assessed.

Lunar phases were expressed as the percentage of the moon daily illuminated (eg, 0% at new moon and 100% at full moon), while the daily distance of the moon from the Earth was measured in kilometers. Holter monitors were evaluated for the average number of supraventricular extrasystoles (SVESs) per hour (total number of SVESs divided by the duration of recording in hours) and for the presence of atrial fibrillation or atrial flutter.

Categorical variables are presented as counts (percentages), and continuous data are presented using median values. Chi-squared goodness-of-fit tests were conducted to determine differences in the incidence of arrhythmias, with *P* ≤ .05 considered statistically significant. The data that support the findings of this study are available upon request.

## Results

During the study period (2012–2020), a total of 58,230 patient presentations were recorded in our ER. Of the patients, 40.3% were women; the median age was 71.5 years (standard deviation [SD], 23.1 years), and the median weight was 78 kg (SD, 33.1 kg). Out of these, 9825 (16.9%) presentations were coded with one of 45 different rhythmological diagnoses **(Table 1)**. The distribution of cardiac arrhythmias did not significantly differ from that of lunar phases during these years (*P* =. 74). Neither subgroups of arrhythmias based on pathophysiological mechanisms (re-entry vs. ectopy) nor individual arrhythmias showed significant differences. For instance, atrial fibrillation (n = 6507, 66% of rhythmological diagnoses) exhibited a uniform distribution across lunar phases (*P* = .85). This also applied to complaints that could be attributed to arrhythmias, such as the sensation of rapid heartbeat and palpitations. Patient sex, age, and weight did not influence the distribution concerning moon illumination. There were no significant differences between the different years.

**Table 1:** The Most Frequent Rhythmological Diagnoses/Groups of Diagnoses

Atrial fibrillation (paroxysmal/persistent/permanent)	6507
Atrial flutter (all types)	1285
Typical flutter	633
Supraventricular tachycardias	244
Ventricular extrasystoles	349
Ventricular tachycardias	292
Supraventricular extrasystoles	54
Other (eg, unclear origin, unclassified, symptom description)	1094

When examining the correlation between the incidence of cardiac arrhythmias and the distance of the moon from the Earth, we found no significant differences in distribution (*P* = .99). A subgroup analysis revealed no correlation concerning sex (*P* = .79), the arrhythmia type, or the patient’s age. However, in a subgroup of underweight patients weighing <55 kg (n = 461), a statistically significant difference was observed. This group presented to the ER with arrhythmias more frequently on days with a relatively greater distance from the moon (385,080 vs. 386,750 km; *P* = .02).

To verify these findings, we investigated a second patient cohort. All underweight patients (<55 kg) presenting to the ER between 2017 and 2020, regardless of the presentation reason, were included (n = 514) and screened for existing Holter electrocardiogram (ECG) recordings. Out of these, 102 patients had Holter ECG recordings over 195 days. These recordings were re-evaluated, assessing the average number of SVESs per hour and episodes of atrial fibrillation/atrial flutter. We observed a uniform burden of extrasystoles irrespective of the moon’s distance from the Earth (Pearson correlation coefficient [*r*] = 0.006; *P* = .94), with no increase in extrasystoles at greater distances. Atrial fibrillation and atrial flutter events occurred on 14.3% of days (n = 28) and showed no dependence on distance.

## Discussion

Cardiac arrhythmias, due to their paroxysmal nature, can remain undiagnosed for extended periods and have potentially life-threatening consequences. Their occurrence cannot be predicted, and they often cannot be induced in electrophysiological studies. Improved timing of diagnostic procedures, such as Holter monitoring and electrophysiological studies, could lead to quicker diagnoses and prevent complications. A correlation with lunar phases or the distance of the moon from the Earth might aid in adjusting diagnostics for better sensitivity. Additionally, some patients believe in such a connection and report occurrences of their arrhythmias, eg, during full or new moons.

Previous studies suggest that lunar phases can be associated with the incidence of acute cardiovascular and neurological disorders, including myocardial infarction,^1,2^ hemorrhagic stroke,^3^ gastrointestinal bleeding,^4^ seizures,^5^ and cardiopulmonary resuscitations.^6^ However, others did not find an association of moon phases with the incidence of myocardial infarction,^7^ seizures,^8^ or ischemic stroke.^3^ Regarding cardiac arrhythmias, there are no systematic analyses available; only a single report on a patient where a lunar periodicity was observed has been published.^9^ The moon may also impact other bodily functions, such as blood pressure and sleep,^10^ that can subsequently predispose an individual to cardiac arrhythmias.

Our retrospective analysis of all emergency department presentations between 2012 and 2020 showed no correlation between lunar phases and the occurrence of arrhythmias. This held true for all arrhythmias collectively, as well as for subgroups based on pathophysiological mechanisms—namely, ectopy or re-entry. Even the most commonly occurring atrial fibrillation and atrial flutter were separately examined and showed no correlation. Age, sex, and weight did not play a role in these findings.

Considering that the effects of lunar phases, ie, moonlight exposure, depend on modern human lifestyle factors (residing in homes with shutters and curtains) and weather conditions (cloud cover, etc.), we proposed investigating the potential correlation with the more independent factor of the distance between the moon and the Earth. However, our analysis once again showed no correlation between the moon–Earth distance and arrhythmias, regardless of the arrhythmia type, sex, and age. An extensive subgroup analysis of the different weight groups was conducted as the mass (weight) could influence the gravitational force exerted by a celestial body. Only in a small subgroup of underweight patients (<55 kg) did a statistical significance emerge, with increased arrhythmias on days when the moon was farther from the Earth.

To examine the possible random component of this difference in patients weighing <55 kg, we analyzed all available Holter monitoring from underweight patients (<55 kg) who presented to our emergency department between 2017 and 2020, regardless of the reason for presentation. Before 2017, our clinic used a different analysis program. We examined the identified 195 24-h long-term ECGs for SVESs and atrial fibrillation/atrial flutter. The reason for selecting SVESs is that they are triggers for almost all supraventricular arrhythmias and are less susceptible to misinterpretation due to artifact interference based on morphological criteria. The average hourly burden of SVESs showed a uniform distribution on all days, regardless of the moon–Earth distance. Similarly, atrial fibrillation/atrial flutter episodes showed no preference for greater or smaller distances. This leads us to conclude that the initially observed difference was more likely a chance finding in an extensive subgroup analysis.

The limitations of our study include its monocentric analysis at only one site with specific geographical and weather conditions, as well as the relatively small number of underweight patients weighing <55 kg. In this group, older women were overrepresented in our analysis. Larger registries could potentially validate the results.

## Conclusions

Moon phases and the distance of the moon from the Earth did not influence the frequency of presentations with rhythmological complaints and diagnoses in the emergency department in our monocentric analysis. This held true regardless of age, sex, or the type of arrhythmia. Contrary to the belief of some patients, the moon does not appear to trigger arrhythmias. The moon cannot assist in expediting the diagnosis of paroxysmal arrhythmias.
